# Perinatal arterial ischemic stroke diagnosed in infants receiving therapeutic hypothermia for hypoxic-ischemic encephalopathy

**DOI:** 10.1038/s41390-024-03531-7

**Published:** 2024-08-27

**Authors:** Fernando F. Gonzalez, Sarah E. Monsell, Marie-Coralie Cornet, Hannah Glass, Jessica Wisnowski, Amit Mathur, Robert McKinstry, Yi Li, Tai-Wei Wu, Dennis E. Mayock, Patrick J. Heagerty, Sandra E. Juul, Yvonne W. Wu

**Affiliations:** 1 Department of Pediatrics; UCSF Benioff Children’s Hospital, University of California San Francisco, San Francisco, CA USA; 2 Department Biostatistics, University of Washington, Seattle, WA USA; 3 Department of Neurology and Weill Institute for Neuroscience, University of California San Francisco, San Francisco, CA USA; 4 Department of Radiology, Keck School of Medicine, University of Southern California, Los Angeles, CA USA; 5 Department of Pediatrics, Saint Louis University School of Medicine, St. Louis, MO USA; 6 Mallinckrodt Institute of Radiology, Washington Univ School of Medicine, St. Louis, MO USA; 7 Department of Radiology and Biomedical Imaging, University of California San Francisco, San Francisco, CA USA; 8 Department of Pediatrics, Children’s Hospital Los Angeles, University of Southern California Keck School of Medicine, Los Angeles, CA USA; 9 Department of Pediatrics, Division of Neonatology, University of Washington School of Medicine, Seattle, WA USA

## Abstract

**Background:**

Both perinatal arterial ischemic stroke (PAIS) and hypoxic-ischemic encephalopathy (HIE) can present with neonatal encephalopathy. We hypothesized that among infants undergoing therapeutic hypothermia, presence of PAIS is associated with a higher risk of seizures and a lower risk of persistent encephalopathy after rewarming.

**Methods:**

We studied 473 infants with moderate or severe HIE enrolled in the HEAL Trial who received a brain MRI. We defined PAIS as focal ischemic infarct(s) within an arterial distribution, and HIE pattern of brain injury as central gray, peripheral watershed, or global injury. We compared the risk of seizures (clinically suspected or electrographic), and of an abnormal 5-day Sarnat exam, in infants with and without PAIS.

**Results:**

PAIS was diagnosed in 21(4%) infants, most of whom (16/21, 76%) also had concurrent HIE pattern of brain injury. Infants with PAIS were more likely to have seizures (RR 2.4, CI 2.8–3.3) and persistent moderate or severe encephalopathy on 5-day Sarnat exam (RR 2.5, 95% CI 1.9–3.4).

**Conclusion:**

Among infants undergoing therapeutic hypothermia, PAIS typically occurs with concurrent HIE pattern brain injury. The higher rate of encephalopathy after rewarming in infants with PAIS may be due to the frequent co-existence of PAIS and HIE patterns of injury.

## Introduction

Neonatal hypoxic-ischemic encephalopathy (HIE) and perinatal arterial ischemic stroke (PAIS) are the two most common causes of brain injury in the term newborn, affecting as many as 1–3 per 1000 live births in high resource settings for HIE and 1 in 1600 live births for stroke.^1–4^ HIE commonly presents with early signs of clinical encephalopathy, and current standard of care for infants with moderate to severe HIE in high resource settings is to initiate therapeutic hypothermia within 6 h of birth as studies consistently demonstrate a reduction in death or neurodevelopmental disability among treated infants.^5^

PAIS can cause significant life-long morbidity in survivors.^6^ The clinical signs of PAIS are similar to that of HIE, namely neonatal encephalopathy and seizures.^7,8^ Although most infants with PAIS have no or minimal encephalopathy at birth but develop seizures or other neurologic symptoms hours or days after delivery, some infants with PAIS will present with encephalopathy early and qualify for therapeutic hypothermia.^9–11^

In a large multicenter cohort of infants who met criteria for moderate or severe HIE undergoing therapeutic hypothermia, we set out to determine the frequency of PAIS diagnosed on MRI and to correlate the presence of PAIS with maternal and infant clinical characteristics. We hypothesized that presence of PAIS is associated with a higher risk of seizures. We further hypothesized that infants with PAIS would have a lower rate of persistent moderate to severe encephalopathy after rewarming, since infants with HIE typically present with more severe encephalopathy than infants with PAIS. Finally, we explored whether presence of PAIS is associated with differences in neurodevelopmental outcomes at 2 years of age.

## Methods

This is an ancillary study of the High-dose Erythropoietin for Asphyxia and Encephalopathy Trial (HEAL, NCT02811263), a phase III randomized, double-blinded, placebo-controlled trial that evaluated the efficacy of erythropoietin combined with therapeutic hypothermia for neonatal HIE.^12^ Five hundred newborns with moderate or severe HIE and undergoing therapeutic hypothermia were enrolled at 17 tertiary care centers, receiving five doses of either Epo or placebo intravenously during the first week after birth. Inclusion criteria were (1) ≥36 weeks gestation; (2) one or more of the following markers of perinatal depression: Apgar <5 at 10 min, need for resuscitation at 10 min, or pH <7.00 or base deficit ≥15 from cord blood or blood sample within the first hour of life; and (3) standardized Sarnat neurological exam indicating moderate or severe encephalopathy between 1–6 h following birth.^13^ Exclusion criteria included genetic or congenital abnormalities, birth weight <1800 grams, head circumference <30 cm, hematocrit >65%, hypoxic-ischemic event occurring only after birth, and redirection of care being considered due to moribund condition. The study was approved by the institutional review board at each participating center, and written informed consent was obtained from the parents or guardians.

Because the diagnosis of PAIS is made on neuroimaging, the current study includes all infants enrolled in HEAL who received a neonatal brain MRI. Brain MRIs were performed after re-warming at 4–6 days (i.e., 96–144 h) of age when possible using a standardized protocol that was harmonized across 9 different 3 T MR scanners and 21 enrolling hospitals as previously described.^14^ The HEAL MRI protocol included conventional T1, T2, and diffusion-weighted sequences.

Three independent readers (RM, JW, AM) reviewed the MRI images to determine the presence of PAIS^14^ with discrepancies resolved via consensus. We defined PAIS as discrete focal region(s) of cytotoxic injury within an arterial distribution which appear distinct and different from HIE patterns of brain injury. We defined HIE pattern brain injury as any of the following patterns: central gray, peripheral watershed, or global (>75% of the cerebrum) injury. Using a validated scoring system we calculated total brain injury scores by summing the extent of injury (i.e., none = 0; <25% = 1; 25–50% = 2; >50% = 3) seen on T1, T2, and apparent diffusion coefficient (ADC) images in eight regions of the brain: caudate, putamen/globus pallidus, thalamus, posterior limb of the internal capsule, cortex, white matter, brainstem, and cerebellum.^15,16^

Neonatal seizures were defined as either clinically suspected seizures or electrographic seizures that were noted on clinical EEG reports. The initial EEG background pattern observed during the first 24 h of age was classified as normal, excessively discontinuous, or severely abnormal (i.e., burst suppression, low voltage, or status epilepticus) as recommended by the American Clinical Neurophysiology Society.^17^ To determine the EEG background, a neonatologist or child neurologist reviewed clinical EEG reports when available, and amplitude-integrated EEG (aEEG) reports if an EEG had not been performed (15/478; 3%). After independently scoring ten percent of all EEG reports, the two EEG reviewers demonstrated substantial interrater agreement (weighted kappa 0.77).

Maternal demographics, labor characteristics and delivery complications were collected during the neonatal hospitalization. The initial severity of HIE was determined by a baseline Sarnat exam performed at 1–6 h of age as previously described.^12^ The severity of encephalopathy after rewarming was determined by the day-5 Sarnat exam, i.e., a repeat Sarnat exam performed in all infants at 4–5 days of age. We defined end-organ injury as any of the following: liver injury (AST > 100 IU/L), kidney injury (creatinine > 1.5 mg/DL), disseminated intravascular coagulopathy (DIC, INR > 2.0), thrombocytopenia (platelet level < 100), intubation, extracorporeal membrane oxygenation (ECMO), or hypotension requiring vasopressor treatment. Neonatal death was defined as death ≤28 days of age.

We explored the relationship between PAIS and the outcome of death or neurodevelopmental impairment (NDI) at two years (i.e., 22–36 months) of age. Neurodevelopmental impairment was defined as any of the following: (1) cerebral palsy; (2) modified Gross Motor Function Classification System (GMFCS) >=1 (i.e., unable to walk 10 steps independently); or (3) cognitive score <90 on Bayley Scales of Infant Toddler Development, third edition (BSID-III). Cerebral palsy was determined by a validated and standardized neurologic examination. Other exploratory outcomes included cerebral palsy and BSID-III cognitive, language and motor scores among survivors at age 2 years.

We evaluated associations between PAIS and clinical characteristics using Boschloo’s non-parametric test of proportions for binary characteristics using the ‘exact2x2’ package in R.^18–20^ The Wilcoxon Rank Sum test was used to compare continuous outcomes. Binary neurodevelopmental outcome and clinical characteristics were compared using relative risks (RRs) with Wald-based 95% confidence intervals (CIs) when cell frequencies were all 5 or above and with the small sample correction when frequencies were less than 5 using the ‘epitools’ package in R.^21^ Continuous outcomes were summarized using medians and interquartile range and confidence intervals were constructed using the Bootstrap method.^22^ Rate ratio confidence intervals were calculated using the exact mid-p method.^21^ Median difference confidence intervals were calculated using the Bootstrap method. All p-values and confidence intervals used a two-tailed significance level of *p* < 0.05, with Holm correction for multiple comparisons only where indicated. All presented analyses are unadjusted. Missing data are noted in the tables. Statistical analyses were performed using R software, version 4.2.3.

## Results

**Fig. 1:**
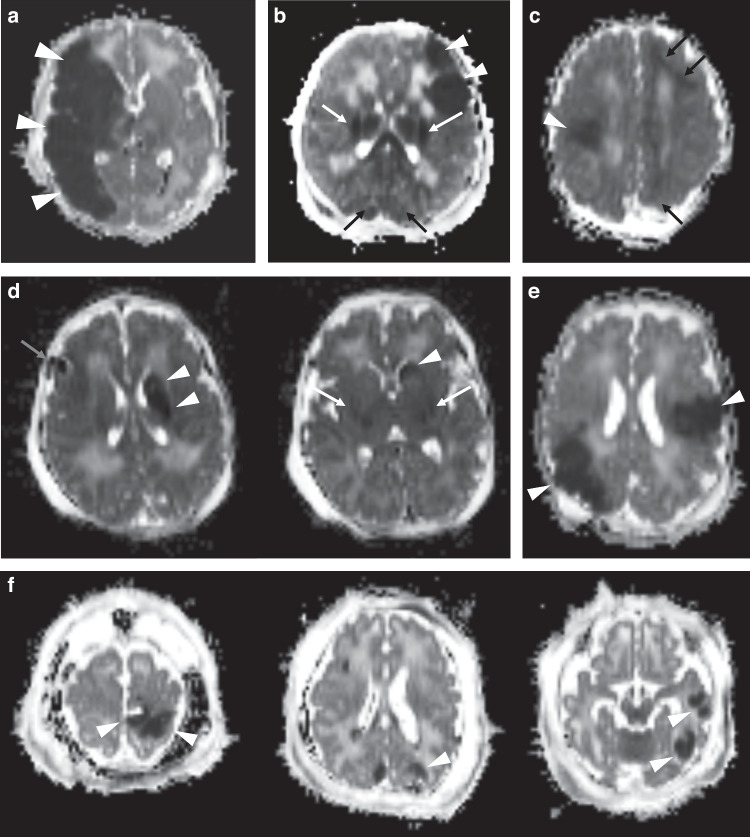
Axial Apparent Diffusion Coefficient (ADC) maps of six patients with perinatal arterial ischemic stroke (PAIS). **a** Infant with reduced diffusion (arrowheads) compatible with PAIS involving the entire right MCA territory. No background features of HIE were present. **b** Infant with left MCA territory focal reduced diffusion (arrowheads) compatible with embolic PAIS. Concomitant HIE pattern brain injury is also present, with reduced diffusion involving the thalami and cortical spinal tracts (white arrows). Additional mild reduced diffusion involving bilateral parietal occipital cortex and subcortical white matter (black arrows), as can be seen with a HIE peripheral watershed pattern of injury. **c** Infant with focal reduced diffusion in right MCA territory (arrowheads) compatible with PAIS and concordant peripheral HIE brain injury pattern (black arrows). **d** Infant with reduced diffusion in left caudate (arrowheads) compatible with PAIS and concordant central HIE pattern (white arrows, lateral putamen) and focal hemorrhage (gray arrow). **e** Infant with reduced diffusion in MCA territory, bilaterally, compatible with multiple PAIS. **f** Infant with multiple focal areas of reduced diffusivity in ACA and MCA territories, bilaterally, compatible with multiembolic PAIS.

Among 500 infants with moderate or severe HIE enrolled in the HEAL Trial, 473 (95%) received a brain MRI. Of those infants who received a brain MRI, 21 (4.4%) were diagnosed with PAIS and 218 (46%) with HIE pattern brain injury. A total of 16 (3.4%) had both PAIS and HIE pattern brain injury on brain MRI (Fig. 1). Specific MRI findings organized by hemisphere and vascular territory are presented in Supplemental Table 1.

Infants with and without PAIS had similar rates of maternal complications and delivery complications (Table 1). The frequency of severe HIE on the baseline Sarnat exam at 1–6 h of age, as well as the rate of low Apgar scores and the median pH and median base deficit, were also similar between the two groups (Table 1). We did not observe any significant difference in Sarnat encephalopathy severity between day 1 and day 5 in PAIS infants compared to PAIS + HIE infants [0/5 severe on day 1 to 1/5 severe on day 5 for PAIS only, 4/16 day 1 to 6/16 severe for PAIS + HIE; *p* = 0.32]. In addition, there were no genetic abnormalities in infants with either PAIS alone or PAIS + HIE.

**Table 1:** Among infants undergoing therapeutic hypothermia for HIE, comparison of maternal and baseline infant clinical characteristics between those with and without PAIS.

	Total *n* = 473 *n* (%)	PAIS *n* = 21 *n* (%)	No PAIS *n* = 452 *n* (%)	*p*-value
Maternal characteristics				
Maternal race				0.57^a^
White	336 (71)	14 (67)	322 (71)	
Black	62 (13)	5 (24)	57 (13)	
Asian	31 (7)	1 (5)	30 (7)	
Multiple/Other	16 (3)	0 (0)	16 (4)	
Unknown	28 (6)	1 (5)	27 (6)	
Maternal age ≥ 35	121 (26)	3 (14)	118 (26)	0.25
Primiparous	270 (57)	14 (67)	256 (57)	0.38
Obesity	85 (18)	4 (19)	81 (18)	0.91
Gestational diabetes	56 (12)	1 (5)	55 (12)	0.40
Pregnancy-induced hypertension	54 (11)	3 (14)	51 (11)	0.71
Preeclampsia or eclampsia	43 (9)	2 (10)	41 (9)	>.99
Any sentinel event	188 (40)	5 (24)	183 (40)	0.13
Placental abruption	67 (14)	0 (0)	67 (15)	0.05
Uterine rupture	24 (5)	0 (0)	24 (5)	0.51
Tight nuchal cord	65 (14)	3 (14)	62 (14)	0.97
Prolapsed cord	22 (5)	0 (0)	22 (5)	0.57
Shoulder dystocia	30 (6)	2 (10)	28 (6)	0.62
Emergency cesarean	298 (63)	14 (67)	284 (63)	0.75
Clinical chorioamnionitis	63 (13)	2 (10)	61 (13)	0.74
Placental disease				
Acute only	62 (20)	4 (33)	58 (20)	0.29
Chronic only	66 (22)	0 (0)	66 (23)	0.06
Acute and chronic	131 (43)	6 (50)	125 (43)	0.64
No abnormality	45 (15)	2 (17)	43 (15)	0.89
Infant characteristics				
Birth weight g, median (IQR)	3327 (2990, 3740)	3275 (3160, 3590)	3330 (2990, 3741)	0.87
Gestational age week, median (IQR)	39.1 (38.0, 40.3))	39.3 (38.6, 40.6)	39.1 (38.0, 40.3)	0.48
Male sex	261 (55)	13 (62)	248 (55)	0.54
5 min Apgar < 5^b^	381 (82)	18 (86)	363 (82)	0.76
10 min Apgar < 5^b^	256 (59)	15 (75)	241 (58)	0.15
Delivery room intubation	328 (69)	17 (81)	311 (69)	0.25
Worst pH^b^, median (IQR)	6.9 (6.8,7.0)	6.9 (6.8,7.1)	7.0 (6.8,7.0)	0.48
Base deficit, median (IQR)	−18.0 (−22.3, −14.0)	−18.0 (−22.5, −15.0)	−18.0 (−22.2, −14.0)	0.63
Severe HIE on baseline Sarnat exam	101 (21)	7 (33)	94 (21)	0.19
Erythropoietin treatment	11 (52)	231 (51)	1.0 (0.7, 1.6)	0.92

^a^White vs. non-white among those with known race.

^b^9 infants without PAIS were missing data on 5-min Apgar score. 1 infant with PAIS and 40 without PAIS were missing data on 10-min Apgar score. 1 infant with PAIS and 35 without PAIS were missing data on minimum pH. 2 infants with PAIS and 46 without PAIS were missing data on base deficit.

Table 2 compares the rates of neonatal complications in infants with and without PAIS. As expected, infants with PAIS were more likely to have seizures (20/21, 95% vs. 152/452, 34%; RR 2.4, CI 2.8, 3.3). The EEG background was also more likely to be excessively discontinuous or severely abnormal in infants with PAIS than in those without PAIS.

**Table 2:** Among infants who received therapeutic hypothermia, a comparison of neonatal complications between those with and without PAIS.

	PAIS present *n* = 21 *n* (%)	PAIS absent *n* = 452 *n* (%)	Relative Risk (95% CI)
Seizures	20 (95)	152 (34)	2.4 (2.8, 3.3)
Anti-seizure medications	20 (95)	177 (40)	3.0 (2.2, 4.1)
EEG background in first 24 h			
Normal	3 (15)	180 (41)	Reference
Excessively discontinuous	9 (45)	158 (36)	1.6 (1.1, 2.3)
Severely abnormal	8 (40)	96 (22)	2.1 (1.4, 3.1)
5-day Sarnat exam^a^			
Normal	1 (4.8)	97 (22)	2.5 (1.9, 3.4)^b^
Mild	5 (24)	225 (50)	
Moderate	11 (52)	82 (18)	
Severe	4 (19)	45 (10)	
End-organ injury			
Liver (AST > 100 IU/L)	17 (85)	288 (67)	1.3 (1.0, 1.6)
Kidney (creatinine >1.5 mg/dL)	2 (10)	45 (10)	0.94 (0.24, 3.6)
DIC (INR > 2.0)	13 (62)	123 (31)	2.0 (1.4, 2.9)
Thrombocytopenia (platelets<100)	3 (14)	10 (2)	5.7 (1.7, 19)
Intubation	17 (81)	311 (69)	1.2 (0.95, 1.5)
ECMO	4 (19)	14 (3)	5.8 (2.1, 16)
Hypotension requiring treatment	14 (67)	149 (33)	2.0 (1.5, 2.8)
Brain MRI findings			
HIE pattern of brain injury			
Central gray	9 (43)	165 (37)	1.2 (0.71, 2.0)
Peripheral watershed	10 (48)	109 (24)	2.0 (1.2, 3.2)
Global injury	2 (10)	33 (7)	1.3 (0.33, 4.9)
At least one HIE pattern of injury	16 (76)	202 (45)	1.7 (1.3, 2.2)
Total brain injury score, median (IQR)	22 (19, 40)	7 (2, 20)	15 (11, 32)^c^
Neonatal death (≤28 days of age)	4 (19)	27 (6)	3.1 (1.2, 8.0)

^a^5-day Sarnat was missing in 3 infants without PAIS. EEG background was missing for 1 infant with and 18 infants without PAIS. Highest AST was missing in 1 infant with PAIS and 21 without PAIS. Highest INR was missing in 58 infants without PAIS. Thrombocytopenia was missing in 16 infants without PAIS.

^b^Relative risk of a moderate or severely abnormal 5-day Sarnat exam compared to normal or mildly abnormal exam.

Most infants with PAIS (16/21, 76%) had presence of both PAIS and HIE pattern brain injury on MRI. Specifically, peripheral watershed injury was more common in infants with PAIS than those without PAIS (Table 2). Infants with PAIS had more neonatal complications than those without PAIS, including end-organ injury such as liver injury, DIC, thrombocytopenia, and hypotension. They were also more likely to have persistent moderate or severe encephalopathy on the 5-day Sarnat exam (15/21, 71% vs. 127/449, 28%; RR 2.5, CI 1.9,3.4) and to have a history of ECMO and to experience neonatal death than those without PAIS (Table 2).

In exploratory analyses, the presence of PAIS was not significantly associated with the risk of death or NDI (13/21 62% vs. 225/433 52%, RR 1.3, 95% CI 0.9, 1.8). Similarly, among survivors, there was no meaningful association between PAIS and motor or cognitive outcomes at two years of age (Table 3).

**Table 3:** Exploratory analyses of 2-year outcomes in infants with and without PAIS.

	PAIS present *n* = 21 *n* (%)	PAIS absent *n* = 452 *n* (%)	Relative Risk (95% CI)^a^
Death or NDI	13 (62)	208 (48)	1.3 (0.91, 1.8)
Cerebral palsy	*n* = 17	*n* = 385	1.7 (0.70, 4.2)
No cerebral palsy	13 (76)	333 (86)	
Cerebral palsy	4 (24)	52 (14)	
Bayley-III^b^	*n* = 17	*n* = 377	Difference in Medians
Cognitive score	90 (85, 95)	90 (80, 100)^c^	0 (−5, 5)
Language score	89 (83, 91)	89 (74, 103)^d^	0 (−8, 5)
Motor score	97 (88,100)	94 (85, 103)^d^	3 (−6, 6)

^a^Relative risk confidence intervals were calculated using the Wald approximation except when cell size<5 in which case the adjustment for small samples was applied.

^b^Median (IQR) and median difference (95% CI).

^c^An additional infant without PAIS was missing a Bayley cognitive score.

^d^An additional 6 infants without PAIS were missing Bayley language and motor scores.

## Discussion

This is the largest study to our knowledge of infants treated with therapeutic hypothermia for HIE who were later diagnosed on brain MRI to have PAIS. We found that 4% of infants with moderate to severe HIE had evidence of PAIS on neuroimaging, and that three-quarters of the infants with PAIS also had concomitant HIE pattern brain injury. We confirmed that seizures were more common in infants with PAIS, but that contrary to our hypothesis, infants with PAIS were more likely to have persistent moderate or severe encephalopathy on the 5-day Sarnat exam than infants without PAIS.

Perinatal stroke occurs during the fetal period or the first 28 days of life and is subdivided into three broad categories: PAIS, cerebral sinovenous thrombosis (CSVT), or hemorrhagic stroke (23). The most common stroke subtype in newborn infants is PAIS, occurring in 80-90% of MRI-diagnosed perinatal strokes.^23,24^ The cause of PAIS is multifactorial; in case-control studies several risk factors have been reported including placental pathology, fetal distress, nulliparity, chorioamnionitis, gestational diabetes, operative delivery, and small for gestational age.^25^ Infants with heritable thrombophilias are also at increased risk, but this is a rare cause of PAIS.^26^ PAIS can result in long-term neurodevelopmental impairments including hemiparetic cerebral palsy, cognitive dysfunction, and epilepsy; outcomes are highly dependent on size and location of the ischemic infarct.^27^

In our cohort, the 4% incidence of PAIS is similar to a previously reported case-control study of infants who received therapeutic hypothermia for presumed HIE.^28–30^ However, others have reported that as many as 31% of cooled infants may have PAIS.^10^ The majority of infants with PAIS present clinically in the first days of life, typically with seizures, apnea, or examination findings of encephalopathy, and often with delayed seizure onset compared to HIE.^24,25^ Previous studies have found that infants with PAIS are more likely to present with seizures, and less likely to have a sentinel event, low Apgar score, or acidosis than infants with HIE10,23. Similarly, a recent study found that infants with HIE were more likely to have an abnormal cord blood gas, a low Apgar score, and seizures when compared to infants with PAIS.^11^

In contrast to previous studies that compared infants with PAIS to infants with HIE, our study only includes infants who met diagnostic criteria for HIE and who received therapeutic hypothermia. This distinction explains why we did not find a difference in the rate of sentinel events, low Apgar scores, or degree of acidosis between infants with and without PAIS. Most infants with PAIS in our cohort also had evidence of HIE pattern brain injury on MRI. This high rate of combined types of brain injury is the likely explanation for why infants with PAIS had a higher rate of persistent moderate to severe encephalopathy on the 5-day Sarnat exam, and were more likely to experience end-organ injury.

While PAIS and HIE are both causes of ischemia-reperfusion injury to the neonatal brain, their underlying mechanisms are quite different. PAIS is caused by a clot that travels from the placenta or cardiovascular system through the open foramen ovale into a major arterial vessel in the brain. In contrast, HIE results from a global reduction of blood flow and oxygen delivery to the brain. In both PAIS and HIE, the brain injury progresses through periods of primary necrotic cell death, secondary energy failure leading to programmed cell death pathways, and a tertiary phase featuring both brain remodeling and neurodegeneration.^31^ Because the exact timing and pathogenesis of both PAIS and HIE are rarely understood in most infants, it is even more difficult to understand why these two different types of neonatal brain injury may occur together. Potential explanations include: (1) the two types of neonatal brain injury share similar risk factors such as chorioamnionitis and placental disease^26,32^; (2) a global hypoxic-ischemic insult leading to HIE may necessitate treatment with ECMO which in itself is a risk factor for stroke,^33,34^ and which was indeed more common in our infants with PAIS; (3) the occurrence of PAIS during labor and delivery may lead to a more difficult and prolonged delivery, which in itself may increase the risk of HIE.

Strengths of this study include the large cohort of infants who met strict clinical criteria for HIE from multiple academic centers nationwide, the central blinded interpretation of MRI, and prospectively collected short-term and long-term outcomes. This study has several limitations. Despite the large number of infants with HIE, only 21 had PAIS, thus limiting our ability to detect statistically significant differences in 2-year outcomes. Most importantly, all infants with PAIS had significant encephalopathy in the first 6 h of age and received a diagnosis of HIE, and therefore our findings do not pertain to infants with later clinical presentations of PAIS, nor to infants who do not meet criteria for therapeutic hypothermia. Our study lacks an unaffected control group and all infants received therapeutic hypothermia; thus, our study can neither examine the risk factors for PAIS nor the efficacy of hypothermia for this condition. Finally, the lack of standardized EEG monitoring for diagnosis of seizures, including timing and location of seizure activity, and the inclusion of clinically suspected seizures are additional limitations.

In conclusion, one in 25 infants who received therapeutic hypothermia for HIE in our cohort were later found to have PAIS on brain MRI. Most infants with PAIS also had HIE pattern brain injury, which likely explains why these infants experienced more severe neonatal complications than those without PAIS. Our study emphasizes the importance of brain MRI in identifying all causes of brain injury in infants undergoing therapeutic hypothermia for HIE.

## Supplementary information

Supplemental Table 1

## Data Availability

We will prepare and share a final research data set that the accepted primary pragmatic trial publication is based upon. The final data set will be structured to maximize future scientific value while protecting patient and health system privacy. The UW DCC will remove or de-identify all 18 HIPAA-specified direct identifiers. The aim of our data sharing policy is to strive for the least restrictive plan possible while providing appropriate protection for participant privacy, health system privacy, and scientific integrity. Within 9 months of the end of the final year of funding, a final study data set will be accessible via a supervised private data enclave managed by the National Institute of Neurological Disorder and Stroke (NINDS) at: https://www.ninds.nih.gov/Current-Research/Research-Funded-NINDS/Clinical-Research/Archived-Clinical-Research-Datasets. The shared data set will contain all data collected under both the HEAL Trial protocol and HEAL ancillary studies. Access will be limited to registered users who submit proposed specific questions or analysis plans and sign a data use agreement according to NINDS guidelines. “Supervised” indicates that individual requests are reviewed to protect the intellectual property rights of the project investigative team by restricting external development of manuscripts using the study data that substantially overlap with those that are already in development by study investigators.
