# Supplementary material for: Perinatal arterial ischemic stroke diagnosed in infants receiving therapeutic hypothermia for hypoxic-ischemic encephalopathy
Source: Pediatr Res. 2024 Aug 27;97(3):1140–6. doi: 10.1038/s41390-024-03531-7 (PMC12055579; doi:10.1038/s41390-024-03531-7)
Supplement: Supplementary file 1 — Supplemental Table 1 [file 41390_2024_3531_MOESM1_ESM.pdf]

Supplemental Table 1. Specific MRI findings of PAIS only (n-=5) and PAIS+HIE (n=16) infants organized by hemisphere and vascular territory of PAIS injury

**Arterial Ischemic Stroke**			**Type/pattern**	**Other injury patterns**
**Hemisphere**	**Vascular Territory**	**Locus**		
Left	Lenticulostriate	Caudate, Putamen	PAIS+HIE	Central, peripheral (acute + subacute); Bilateral subdurals with mass effect
Left	Lenticulostriate	Caudate, Putamen	PAIS+HIE	Central; punctate white matter lesions; cerebellar injury
Left	MCA	PreCG, PostCG	PAIS	
Left	MCA	MFG, IFG, PreCG, PostCG, SMG, STG, Insula	PAIS+HIE	Global HIE
Left	MCA	(1) MFG, Insula<br>(2) PostCG, SMG, AG, STG, MTG	PAIS+HIE	Peripheral, central
Left	MCA	PreCG, PostCG, SMG, AG, STG, MTG, ITG, FG, MOG	PAIS	
Left	MCA	PostCG, SMG	PAIS+HIE	Punctate white matter lesions
Left	PCA	CG, LG, SOG, MOG, IOG	PAIS+HIE	Peripheral
Right	Thalamogeniculate (PCOMM)	Lateral posterior nucleus (thalamus)	PAIS+HIE	Central, punctate white matter lesions
Right	MCA	Inferior PreCG + PostCG; STG; SMG AG	PAIS+HI	Peripheral (subacute)
Right	MCA	Caudate, Putamen, Insula, MFG, IFG, PreCG, PostCG, SMG, AG, STG, MTG, ITG, FG, MOG	PAIS	Also, IVH + venous infarcts in bilateral thalamus
Right	MCA	PreCG, PostCG	PAIS+HIE	Peripheral
Right	MCA	PostCG, SMG, AG, STG, Insula	PAIS+HIE	Peripheral
Bilateral	MCA	(1) Left: PreCG, PostCG<br>(2) Right: STG, MTG, ITG, SMG, AG, MOG	PAIS	
Bilateral	MCA	(1) Left: MFG, IFG, Insula	PAIS+HIE	Global HIE

		(2) Left: PreCG, PostCG<br>(3) Left: STG, MTG, MOG<br>(4) Right: PreCG, PostCG		
Bilateral	Polar (PCOMM)<br>MCA<br>PCA	(1) Left: Anterior + Dorsolateral nuclei (thalamus)<br>(2) Left: MTG<br>(3) Left: AG<br>(4) Left: PC<br>(5) Left: ITG, FG<br>(6) Right: PreCG	PAIS+HIE	Peripheral, IVH
Bilateral	Lenticulostriate<br>Polar (PCOMM)<br>MCA<br>PCA	(1) Left: Caudate<br>(2) Left: SMG, AG, PC<br>(3) Right: Anterior nuc (thalamus)<br>(4) Right: PostCG, SMG, AG, STG, MTG, SOG, CG	PAIS+HIE	Peripheral, IVH, IPH
Bilateral	PCA, MCA	(1) Left: Retrosplenial CingG; PC; CG<br>(2) Right: PreCG<br>(3) Right: STG	PAIS+HIE	Central, Peripheral
Bilateral	PCA	(1) Left: FG + ITG<br>(2) Right: FG	PAIS	
Bilateral	PCA,<br>Polar (PCOMM)	(1) Left: CG, LG<br>(2) Right: Anterior nuc. (thalamus)	PAIS+HIE	Peripheral
Bilateral	PCA, ACA	(1) Left: CingG, PCL<br>(2) Right: CG, LG, PHG	PAIS+HIE	Punctate white matter lesions

Notes: PAIS=arterial ischemic injury on MRI without evidence of HIE; PAIS+HIE=evidence of both arterial ischemic injury and hypoxic-ischemic injury on MRI. ACA = Anterior Cerebral Artery; MCA = Middle Cerebral Artery; PCA = Posterior Cerebral Artery; PCOMM = Posterior Communicating Artery; AG = Angular Gyrus; CG = Cuneus; CingG = Cingulate Gyrus; FG = Fusiform Gyrus; ITG = Inferior Temporal Gyrus; LG = Lingual Gyrus; MOG = Middle Occipital Gyrus, PC = Precuneus; PCL = Paracentral Lobule; PHG = Parahippocampal Gyrus; PreCG = Precentral Gyrus; PostCG = Postcentral Gyrus; STG = Superior Temporal Gyrus; IVH = intraventricular hemorrhage; IPH = intraparenchymal hemorrhage
